# Overexpressed *XRCC2* as an independent risk factor for poor prognosis in glioma patients

**DOI:** 10.1186/s10020-021-00316-0

**Published:** 2021-05-29

**Authors:** Zhendong Liu, Wang Zhang, Xingbo Cheng, Hongbo Wang, Lu Bian, Jialin Wang, Zhibin Han, Yanbiao Wang, Xiaoyu Lian, Binfeng Liu, Zhishuai Ren, Bo Zhang, Zhenfeng Jiang, Zhiguo Lin, Yanzheng Gao

**Affiliations:** 1 Department of Orthopaedics, Henan Provincial People’s Hospital, People’s Hospital of Zhengzhou University, School of Clinical Medicine, Henan University, Zhengzhou, Henan China; 2 Department of Neurosurgery, The First Affiliate Hospital of Harbin Medical University, 23 Youzheng Street, Nangang District, Harbin, 150001 China; 3 Microbiology Laboratory, Henan Provincial People’s Hospital, Zhengzhou, China

**Keywords:** Glioma, *XRCC2*, Oncogene, Prognosis, Biomarker

## Abstract

**Background:**

*XRCC2*, a homologous recombination-related gene, has been reported to be associated with a variety of cancers. However, its role in glioma has not been reported. This study aimed to find out the role of *XRCC2* in glioma and reveal in which glioma-specific biological processes is *XRCC2* involved based on thousands of glioma samples, thereby, providing a new perspective in the treatment and prognostic evaluation of glioma.

**Methods:**

The expression characteristics of *XRCC2* in thousands of glioma samples from CGGA and TCGA databases were comprehensively analyzed. Wilcox or Kruskal test was used to analyze the expression pattern of *XRCC2* in gliomas with different clinical and molecular features. The effect of *XRCC2* on the prognosis of glioma patients was explored by Kaplan–Meier and Cox regression. Gene set enrichment analysis (GSEA) revealed the possible cellular mechanisms involved in *XRCC2* in glioma. Connectivity map (CMap) was used to screen small molecule drugs targeting *XRCC2* and the expression levels of *XRCC2* were verified in glioma cells and tissues by RT-qPCR and immunohistochemical staining.

**Results:**

We found the overexpression of *XRCC2* in glioma. Moreover, the overexpressed *XRCC2* was associated with a variety of clinical features related to prognosis. Cox and meta-analyses showed that *XRCC2* is an independent risk factor for the poor prognosis of glioma. Furthermore, the results of GSEA indicated that overexpressed *XRCC2* could promote malignant progression through involved signaling pathways, such as in the cell cycle. Finally, doxazosin, quinostatin, canavanine, and chrysin were identified to exert anti-glioma effects by targeting *XRCC2*.

**Conclusions:**

This study analyzed the expression pattern of *XRCC2* in gliomas and its relationship with prognosis using multiple datasets. This is the first study to show that *XRCC2*, a novel oncogene, is significantly overexpressed in glioma and can lead to poor prognosis in glioma patients. *XRCC2* could serve as a new biomarker for glioma diagnosis, treatment, and prognosis evaluation, thus bringing new insight into the management of glioma.

**Supplementary Information:**

The online version contains supplementary material available at 10.1186/s10020-021-00316-0.

## Background

Glioma is the most common primary tumor among adult brain tumors and has high mortality and disability rates (Tan 2020). Currently, the treatment of glioma is mainly surgical intervention and postoperative adjuvant chemoradiotherapy (Lim et al. 2018). Although there are many new methods for treating glioma (Pulkkanen and Yla-Herttuala 2005; Kamran 2018; Mahmoudi 2019), the prognosis of glioma patients remains unsatisfactory. Targeted therapy attacking specific genes within the tumorous cells may provide new ways to kill glioma cells and improve the prognosis of patients. Therefore, it is vital to find new and effective molecular targets for the diagnosis and treatment of glioma.

In recent years, research on molecular targeted therapy has proliferated greatly, and genes related to glioma diagnosis, treatment, and prognosis have been screened and identified. For instance, the *B-RAF* proto-oncogene encoded protein has a regulatory role in the *MAPK*/*ERK* signaling pathway, which regulates glioma cell division and differentiation (Lassaletta 2017); *KRAS* mutations are associated with malignant progression of glioma (Fioravanzo 2019); the zinc finger E-box binding homeobox 1 can inhibit the transcription of interleukin-2 and inhibit the invasion and epithelial-mesenchymal transition of glioma cells (Siebzehnrubl 2013). Although these studies have enriched the molecular pool related to glioma diagnosis and treatment, the prognosis of glioma patients is yet to be significantly improved, which may be due to the fact that the malignant progression of gliomas is triggered by a variety of complex factors rather than by a single oncogene. Therefore, more genes need to be screened and identified to enrich the molecular pool for glioma diagnosis and treatment, thus bringing hope for improving the prognosis of glioma patients.

The malignant transformation of a normal cell is the result of multiple factors, among which DNA double-strand breaks (DSBs) are the most lethal form of cell damage (Ceccaldi et al. 2016; Hoppe et al. 2018; Frappart 2009). As one of the repair methods for DSBs, homologous recombination plays an important role in maintaining the stability of chromosomes and inhibiting the infinite replication of cells; the dysfunction of this repair method can increase the risk of cancer (Hoppe et al. 2018; Chen 2018; Hallam et al. 2015). It is well known that *RAD51*, a DNA repair protein, plays a central role in regulating the homologous recombination repair process of DSBs (Shen 2018; Cruz 2018). Moreover, studies have shown that after ionizing radiation exposure, *RAD51* participates in cell cycle changes in glioblastoma stem cells (Tachon 2018). Furthermore, Ohba et al. found that *RAD51*-mediated homologous recombination showed an increasing trend in isocitrate dehydrogenase 1(*IDH1)* -mutant gliomas (Ohba et al. 2014). *XRCC2*, a homologous gene of *RAD51*, can form a complex with three *RAD51* paralogs (*RAD51B*, *RAD51C*, and *RAD51D*), and it participates in homologous recombination by encoding members of the RECA/RAD51-related protein family (Baldock 2019; Andreassen and Hanenberg 2019). Most studies consider *XRCC2* to be a breast cancer susceptibility gene and have demonstrated that it plays an important role in its pathophysiology (Kleibl and Kristensen 2016; Lin 2011). Previous studies have also shown that *XRCC2* is abnormally expressed in a variety of tumors, including rectal and breast cancers, and that it participates in many cell signaling pathways (Chen 2018; Andreassen and Hanenberg 2019; Xu 2014; Bashir et al. 2014). In addition, *XRCC1* has been reported as one of the possible genes involved in glioma prognosis (Jiang 2013). However, the role and expression pattern of *XRCC2* in glioma have not been elucidated so far.

Currently, computational research has opened new avenues for advanced research on cancer management. Many recent reports have used in silico approaches to predict the potential role of numerous genes and proteins in the development of different types of cancer (Andreassen and Hanenberg 2019; Khan et al. 2017; Li 2020). Therefore, we attempted to clarify the expression pattern of *XRCC2* in glioma through a joint analysis of multiple databases, to explore the correlation between the expression pattern of *XRCC2* and the clinical and molecular characteristics of glioma, and to reveal the value of *XRCC2* in the prognostic evaluation of glioma. Overall, we aimed to identify a novel therapeutic target for glioma and to contribute to the enlargement of the molecular pool for glioma diagnosis and treatment.

## Materials and methods

### Data collection

Gene Expression Profiling Interactive Analysis 2 (GEPIA2, http://gepia2.cancer-pku.cn/) integrates RNA-seq data from The Cancer Genome Atlas (TCGA) and Genotype-Tissue Expression (GTEx) databases (Tang et al. 2019; Consortium GT 2015). We used GEPIA2 to preliminarily explore the expression of *XRCC2* in different cancers and their corresponding normal tissues, including 163 glioblastoma samples and 207 normal brain tissue samples. Since the database is an online analysis platform, the expression level of *XRCC2* in different tumors can be obtained by inputting *XRCC2* in the search page.

The Chinese Glioma Genome Atlas (CGGA, http://www.cgga.org.cn/) database contains thousands of glioma gene expression data and the corresponding clinical information. We downloaded 1018 mRNA sequencing and 301 mRNA microarray data from the CGGA database, of which the mRNA sequencing and mRNA microarray data with complete clinical information were 748 and 268, respectively. The original data in the database has been standardized, so after downloading the original data, the expression of *XRCC2* in samples may be extracted for further analysis.

TCGA (https://portal.gdc.cancer.gov/) is currently the most significant public database for cancer research. We downloaded 698 glioma transcriptome data from the TCGA database (Workflow Type: HTSeq-FPKM), and the remaining 653 data after the removal of incomplete clinical information were used as validation data sets. As the downloaded data types were standardized, there was no need for further processing, only the expression level changes of *XRCC2* in various tissue samples were extracted for further analysis.

Further, we downloaded five glioma datasets from the Gene Expression Omnibus (GEO, http://www.ncbi.nlm.nih.gov/geo/) database, including 279 glioma samples for meta-analysis to validate the effect of *XRCC2* on glioma prognosis. They were GSE4412 containing 85 (platform: GPL96), GSE43378 containing 50 (platform: GPL570), GSE74187 containing 60 (platform: GPL6480), GSE50025 containing 34 (platform: GPL13938), and GSE83300 containing 50 samples of glioma patients (platform: GPL6480).

Firstly, the platform information corresponding to the five different datasets was replaced under the command of Perl software. Subsequently, all datasets were normalized one by one using the program in the limma package of R software. Finally, *XRCC2* expression level and survival time and status of corresponding glioma patients were selected for further meta-analysis.

### Cell culture and preparation of glioma samples

The glioma cell lines LN229, A172, U251, and T98, and the human astrocyte (HA) cell line were purchased from Procell Life Science & Technology Co. Ltd (Wuhan, China). Cells were cultured in DMEM high-sugar medium (Procell, PM150210) containing 10% fetal bovine serum (Gibco, lot: 10099-141c) and 1% penicillin–streptomycin mixture in a 37 °C humidified incubator with 5% carbon dioxide.

Twenty-three glioma samples from 11 women and 12 men were included in this study, with an average age of 50.35 years. Of these, six were world health organization (WHO) grade II, five cases were WHO grade III, and 12 were WHO grade IV Table 1. Nine non-glioma tissues served as controls. All tissues were rapidly frozen in liquid nitrogen within 15 min after surgical resection, and all glioma patients were diagnosed by professional pathologists. All patients provided written informed consent in accordance with the Declaration of Helsinki. The study protocol was approved by the Ethics Committee of Zhengzhou University.

**Table 1:** Characteristics of patients with glioma based on clinical samples

Characteristic	Number (%)	Low expression	High expression	P value
Gender				
Female	11 (47.83)	3	8	NS
Male	12 (52.17)	7	5	
Age (year)				
> 50.35	14 (60.87)	7	7	NS
≤ 50.35	9 (39.13)	3	6	
Grade				
WHO II	6 (26.09)	5	1	0.019*
WHO III	5 (21.74)	3	2	
WHO IV	12 (52.17)	2	10	
Primary recurrent state				
Primary	19 (0.83)	8	11	NS
Recurrent	4 (0.17)	2	2	

The high and low XRCC2 expression groups were divided according to the mean expression level of XRCC2. *NS* not significant. *Chi-Square Test

### RNA isolation and real-time quantitative polymerase chain reaction (RT-qPCR)

According to the manufacturer’s instructions, total RNA was extracted using Trizol (Invitrogen, lot: 262306), cDNA was obtained using NovoScript Plus All-in-one 1st Strand cDNA Synthesis SuperMix (gDNA Purge) (Novoprotein, E047), and RT-qPCR was performed using 2x RealStar Green Fast Mixture with ROX (GenStar, A303-05). RNA-specific primer sequences for the internal reference gene *GAPDH* were forward: 5′-AAGAAGGTGGTGAAGCAGG-3′ and reverse: 5′-GTCAAAGGTGGAGGAGTGG-3′. The specific primer sequences of the target gene *XRCC2* were forward: 5′-GAGCACAGACTATCCCAAAG-3′ and reverse: 5′-CAGGCTATCCAATCAAAA-3'.

### Immunohistochemical staining

After deparaffinization and hydration in xylene, graded alcohols, paraffin tissue sections with a thickness of 3 µm were subjected to antigen retrieval using microwave retrieval for 15 min (EDTA, pH 8.0). Then 100 µL of primary antibodies against XRCC2 (ab180752, 1:100 dilution) was dropped onto the tissue sections and incubated overnight at 4 °C. Horseradish peroxidase-labeled IgG polymer (PV6000, Zhongshan Jinqiao Biotechnology, China) as a secondary antibody incubated the tissues for 40 min at room temperature condition. Finally, 3,3′-diaminobenzidine Kit (ZLI-9017, Zhongshan Jinqiao Biotechnology, China) was added as a chromogen, and the sections were evaluated using a light microscope.

### Meta-analysis

We first determined the relationship between *XRCC2* and glioma prognosis by systematically searching the studies of *XRCC2* in glioma in PubMed and Web of Science databases. Since this is the first study to investigate the prognostic role of *XRCC2* in glioma, no published studies relating *XRCC2* with glioma prognosis were obtained from public databases. Therefore, we utilized the meta-analysis to evaluate the overall prognostic significance of *XRCC2* in glioma patients based on eight datasets using R software (version 3.6.3), and data were extracted and selected according to Preferred Reporting Items for Systematic reviews and Meta-Analyses (PRISMA) guidelines (Keerthana and Kumar 2020). The association of *XRCC2* expression with the prognosis of glioma patients was evaluated by calculating the combined hazard ration (HR) and 95% confidence interval (CI). The heterogeneity of the eight datasets was assessed by the Q test (I^2^ statistics). I^2^ < 50% was considered as moderate heterogeneity and a fixed effects model was chosen for combination. Otherwise, random effects model was applied. The Begg and Egger funnel chart was used to assess the possibility of publication bias. However, this part of the content has not been presented separately, because when there are fewer than 10 studies in the Meta-analysis, the results of the publication bias test are considered unreliable (Cumpston 2019; Dalton et al. 2016; Egger et al. 1997). This study was registered with PROSPERO (ID: CRD42021245507).

### Gene set enrichment analysis of XRCC2

GSEA is a very convenient tool for bioinformatics analysis, and it can be used to identify the disease-related signaling pathways in which specific genes participate. We used SVA and limma software packages to normalize and correct data downloaded from the TCGA and CCGA databases. The high and low *XRCC2* expression groups were defined according to the expression level of *XRCC2* in glioma. GSEA 4.0.jar software was used to evaluate the enrichment of signaling pathways. The number of permutations was 1000, and the gene database was associated with Kyoto Encyclopedia of Genes and Genomes (KEGG) cell signaling pathways.

### CMap analysis

Connectivity map (CMap, https://portals.broadinstitute.org/cmap/) was used to explore small molecule drugs targeting *XRCC2* in glioma. We first identified genes that were co-expressed with *XRCC2* based on CGGA RNA-seq data using Pearson correlation analysis. Genes positively and negatively correlated with *XRCC2* were then uploaded to the CMaP online tool, and P < 0.01 and enrichment < 0.8 were used as criteria to identify the small molecule drugs targeting *XRCC2*.

### Statistical analysis

Statistical analysis was performed using R software (version 3.6.3). Wilcoxon test or Kruskal–Wallis test was used to analyze differences in *XRCC2* expression in gliomas with different clinical and molecular characteristics. Kaplan–Meier and Cox regression were used to analyze the impact of *XRCC2* on the prognosis of glioma patients and its value in prognostic diagnosis. Pearson correlation was used to analyze genes co-expressed with *XRCC2* in gliomas. The expression level of *XRCC2* in glioma cells and tissues was evaluated using a relative quantitative method based on the results of three independent experiments. GraphPad Prism 8.0 was used to analyze the differences between groups, and *P* < 0.05 was defined as statistically significant.

## Results

### Data characteristics

Glioma transcriptome data and their corresponding clinical information files were downloaded from the CGGA and TCGA databases, and, after deleting incomplete clinical information, 748 CGGA RNA-seq data Additional file 1: Table S1), 268 CGGA microarray data Additional file 2: Table S2), and 653 TCGA RNA-seq data Additional file 3: Table S3) were included. Each dataset contained at least three detailed data on the clinical characteristics of gliomas. The transcriptome data of thousands of glioma samples with complete clinical information obtained from these three data sets guaranteed the feasibility and reliability of subsequent studies.

### Overexpression of XRCC2 in glioblastoma multiforme

**Fig. 1:**
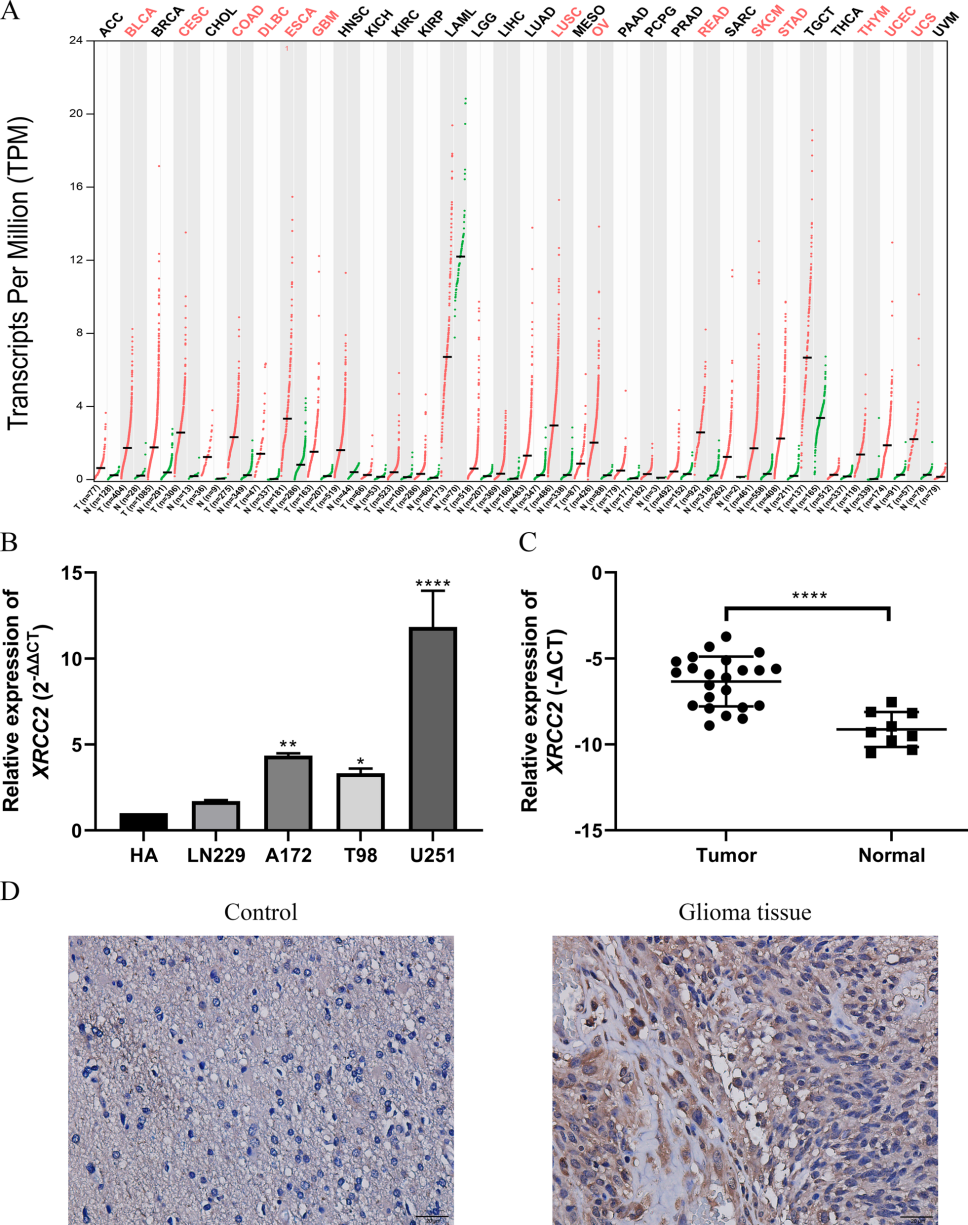
Expression of *XRCC2* in cancers. **A** The expression level of *XRCC2* in cancers based on GEPIA2. **B** The expression level of *XRCC2* in glioma cell lines based on RT-qPCR. **C** The expression level of *XRCC2* in glioma tissues based on RT-qPCR. **D** Immunohistochemical staining results for *XRCC2* in non-glioma and glioma tissues. Brown color represents positive *XRCC2* staining. *P = 0.0108, **P = 0.001, ****P < 0.0001. *GEPIA2* gene expression profiling interactive analysis 2, *RT-qPCR* real-time quantitative polymerase chain reaction

We analyzed the expression of *XRCC2* in various cancers and their corresponding normal tissues using the GEPIA2 online platform. As shown in Fig. 1A, we found that the expression level of *XRCC2* was significantly increased in various cancers, including colon adenocarcinoma, rectum adenocarcinoma, and glioblastoma multiforme (GBM). In order to clarify the expression pattern of *XRCC2* in cancer and its impact on prognosis, we chose GBM as the research object for follow-up study. RT-qPCR results confirmed that the expression level of XRCC2 in glioma cell lines and glioma tissues was significantly higher than that in the corresponding control group Fig. 1B, C. Consistent with the mRNA expression pattern, immunohistochemical staining results indicated XRCC2 expression to be higher in glioma tissues than in benign tissues Fig. 1D.

### Overexpressed XRCC2 predicts poor prognosis in glioma

**Fig. 2:**
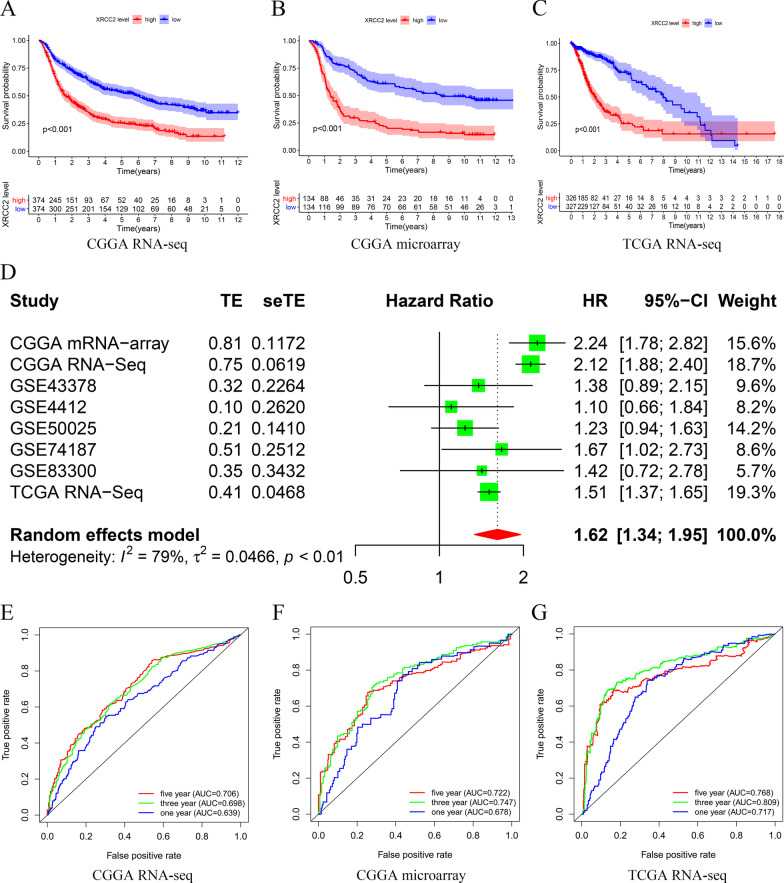
Overexpression of *XRCC2* predicts poor prognosis. **A**–**C** Kaplan–Meier survival curves based on CGGA RNA-seq, CGGA microarray, and TCGA RNA-seq, respectively; **D** Forest plot of high XRCC2 expression with better overall survival in glioma patients from eight datasets; **E**–**G**: ROC curves based on CGGA RNA-seq, CGGA microarray, and TCGA RNA-seq, respectively. *CGGA* Chinese Glioma Genome Atlas, *TCGA* The Cancer Genome Atlas, *ROC* receiver operating characteristic

To understand the impact of *XRCC2* on the prognosis of glioma, we first used Kaplan–Meier analysis to analyze the relationship between the expression level of *XRCC2* and the prognosis of glioma patients based on three different data sets. The results showed that overexpressed *XRCC2* could significantly shorten the survival time of glioma patients (*P* < 0.001, Fig. 2A–C. In the TCGA RNA-seq data set, the survival curves of glioma patients crossed each other, which may be due to the small number of patients in the late follow-up period. Nevertheless, the 5-year and 10-year survival rates of patients with low *XRCC2* expression were significantly higher than those of patients with overexpression of *XRCC2*.

**Fig. 3:**
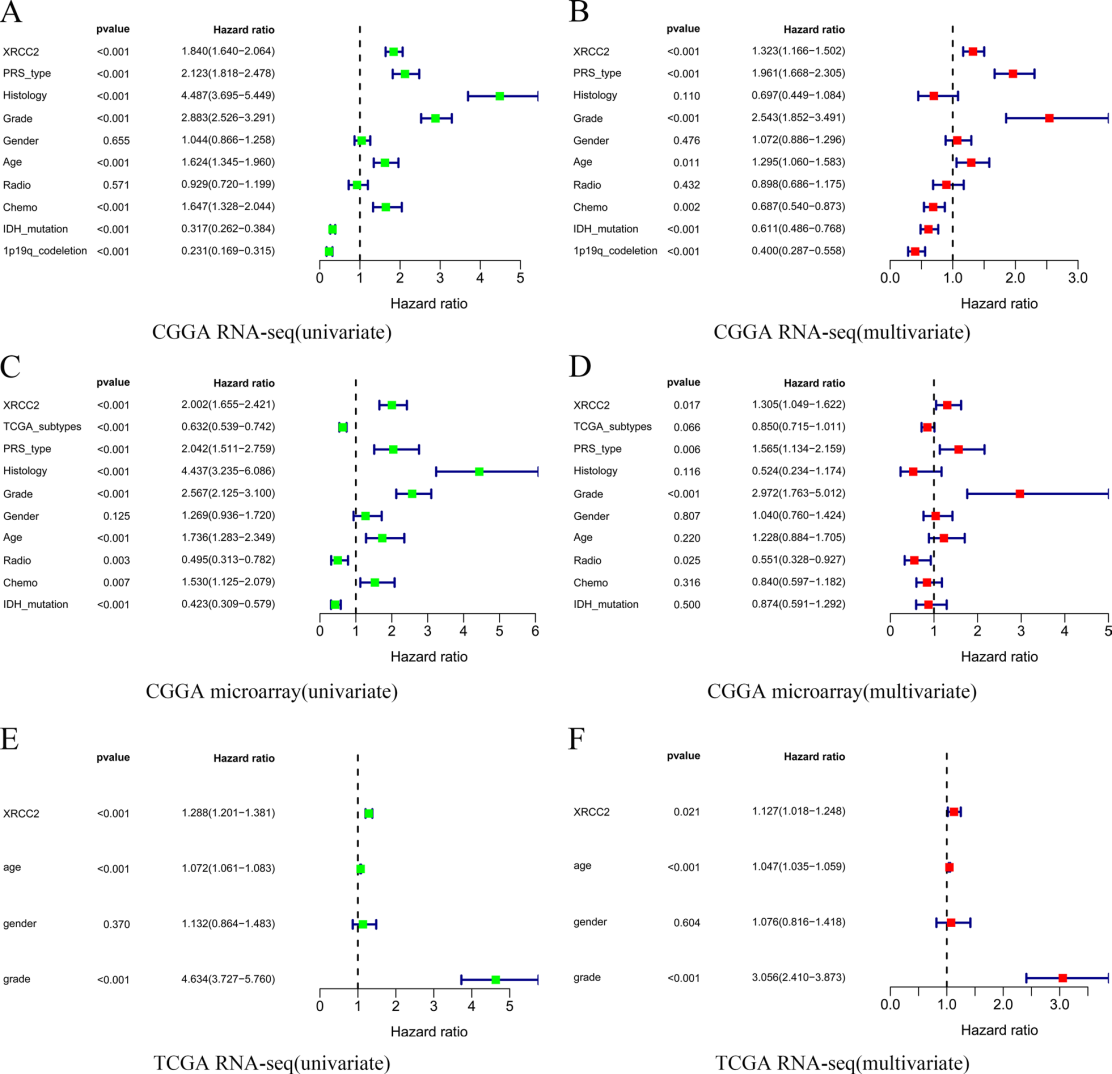
*XRCC2* is an independent risk factor for poor glioma prognosis. **A**, **C**, **E** Results of the univariate analysis based on CGGA RNA-seq, CGGA microarray, and TCGA RNA-seq, respectively; **B**, **D**, **F** Results of the multivariate analysis based on CGGA RNA-seq, CGGA microarray, and TCGA RNA-seq, respectively. *CGGA* Chinese Glioma Genome Atlas; *TCGA* The Cancer Genome Atlas

Since *XRCC2* has not been previously reported in glioma, to observe the pooled effect of high expression of *XRCC2* on patient prognosis, we integrated *XRCC2* gene expression and patient survival information from eight datasets in the GEO, TCGA, CGGA databases, and the results of meta-analysis showed that the pooled HR along with 95% CI for the correlation between highly expressed *XRCC2* and prognosis was 1.62 (1.34–1.95) Fig. 2D, which indicated that *XRCC2* is a risk factor for glioma prognosis. Furthermore, the results of the Cox analysis determined by the area under the receiver operating characteristic (ROC) curve (AUC) values suggested that *XRCC2* is potentially useful for the prognostic assessment of glioma, especially for 5-year outcome (*P* > 0.7, Fig. 2E–G. To further clarify whether *XRCC2* is an independent risk factor for the prognosis of glioma patients, we conducted univariate and multivariate analyses. The results indicated that *XRCC2* acts as an independent factor in the prediction of prognosis of glioma patients, and this result was mutually validated among three different data sets (*P* < 0.05, HR > 1, Fig. 3. Taken together, we believe that the overexpression of *XRCC2* may be considered an independent risk factor of poor prognosis in glioma patients.

### Relationship between XRCC2 expression and clinical features of glioma

**Fig. 4:**
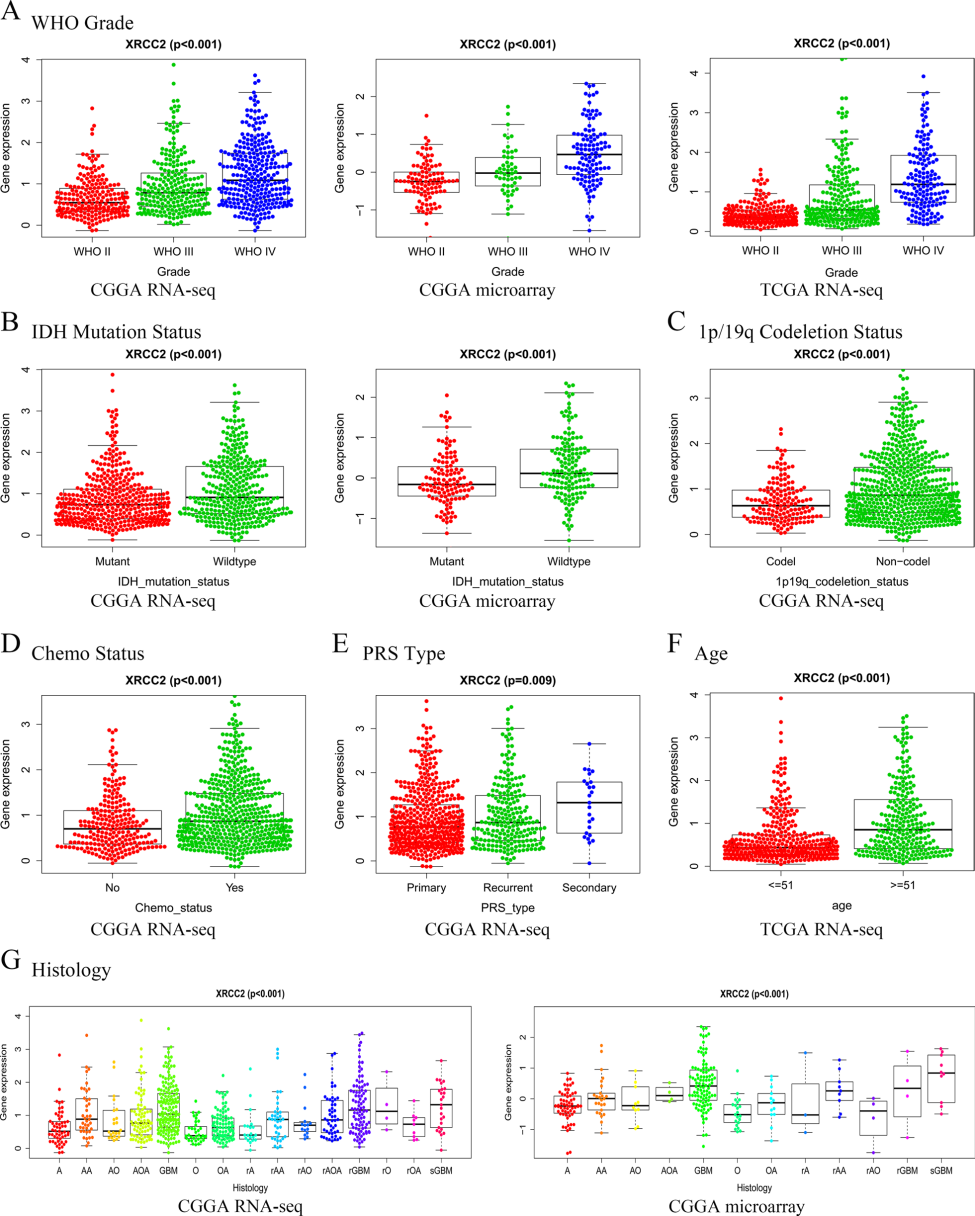
Correlation between high *XRCC2* expression and different clinical features of glioma. **A** Grade; **B** *IDH* mutation presence; **C** *1p/19q* co-deletion presence; **D** Chemo status; **E** PRS Type; **F** Age; **G** Histology. *PRS* primary recurrence and secondary

It is well known that the prognosis of glioma patients is associated with a variety of clinical features, such as glioma grade, primary recurrence status of tumors, and mutations in specific molecules. Since the results of this study suggest that overexpression of *XRCC2* can lead to poor prognosis, we speculated that different clinical features of gliomas may predict differences in *XRCC2* expression. Therefore, we analyzed the expression levels of *XRCC2* in gliomas with different characteristics based on the three different data sets. As shown in Fig. 4A, *XRCC2* has different expression levels in different grades of glioma. Moreover, the expression level of *XRCC2* increased with malignity (*P* < 0.001). Consistently, the expression level of *XRCC2* in recurrent gliomas was higher than that in primary gliomas, and this was maintained for several histological subtypes Fig. 4E, G. Interestingly, the presence of *IDH* mutation and *1p/19q* co-deletion negatively correlated with *XRCC2* expression. As shown in Fig. 4B, C, the expression of *XRCC2* was reduced in gliomas with *IDH* mutation and *1p/19q* co-deletion (*P* < 0.001). Since *IDH* mutation and *1p/19q* co-deletion are currently recognized as predictors of better prognosis in glioma patients, this result indirectly supports the adverse effect of *XRCC2* on the prognosis of glioma. In addition, *XRCC2* has higher expression levels in patients receiving chemotherapy and in elderly patients Fig. 4D, F, *P* < 0.001). In addition, the samples were divided into high expression and low expression groups according to the average value of *XRCC2* expression in the 23 glioma samples. The Chi-Square test showed that the expression level of *XRCC2* in glioma tissues was significantly correlated with WHO grade (*P* = 0.019, Table 1. Simultaneously, we also found that the expression level of *XRCC2* was not significant between different ages and different primary-recurrence status based on the results of clinical sample analysis (*P* > 0.05, Table 1, which might have been because of the relatively small sample size. These results suggest that the expression of *XRCC2* is correlated with clinical features associated with the prognosis of gliomas and that highly expressed *XRCC2* may be involved in the malignant progression of gliomas through different mechanisms.

### GSEA-revealed XRCC2 signaling pathways involved in glioma progression

**Fig. 5:**
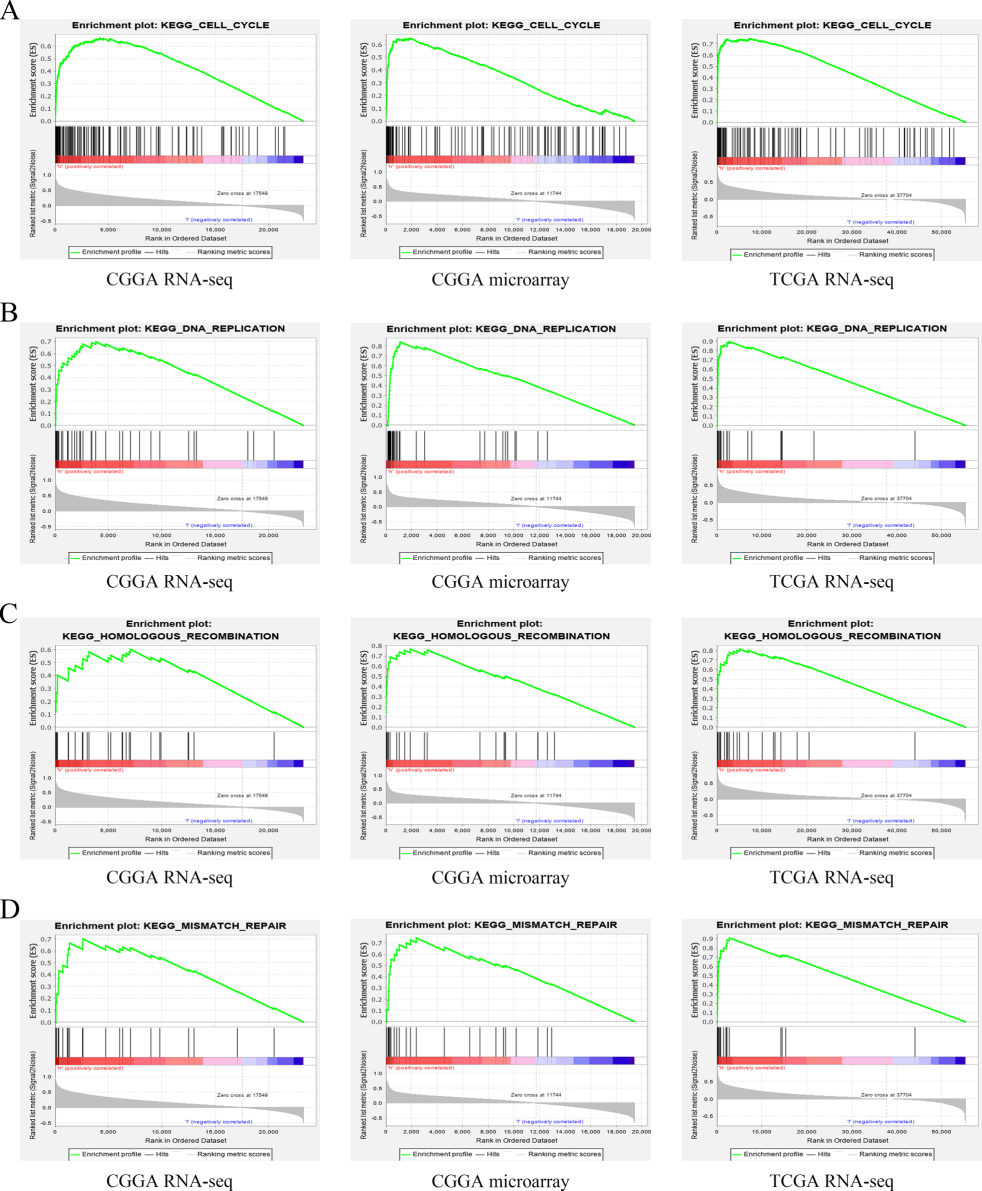
Enrichment results of GSEA based on different datasets. **A** Cell-cycle, **B** DNA-replication; **C** Homologous-recombination; **D** Mismatch-repair. *GSEA* gene set enrichment analysis

To elucidate the cellular mechanisms by which the overexpressed XRCC2 leads to tumoral progression in glioma, we used GSEA to analyze the data from the CGGA and TCGA databases. The signal pathways of cell cycle, DNA replication, homologous recombination, and mismatch repair obtained higher enrichment scores than the rest. Moreover, this result was mutually validated in the three datasets Fig. 5, Table 2). These results suggest that *XRCC2* plays a cancer-promoting role in glioma by participating in different signaling pathways.

**Table 2:** Signal pathways involved in *XRCC2* in glioma based on three datasets

Pathways	CGGA RNA-seq			CGGA microarray			TCGA RNA-seq		
	NES	NOM *P* -value	FDR q-value	NES	NOM *P* -value	FDR q-value	NES	NOM *P* -value	FDR q-value
Cell cycle	1.950	0.000	0.123	1.983	0.004	0.021	2.239	0.000	0.001
DNA replication	1.715	0.029	0.146	1.966	0.000	0.017	2.048	0.000	0.005
Homologous recombination	1.572	0.046	0.178	2.046	0.000	0.015	2.024	0.002	0.004
Mismatch repair	1.715	0.008	0.159	1.797	0.015	0.105	2.076	0.000	0.004

*NES* normalized enrichment score, *NOM* nominal, *FDR* false discovery rate. Gene sets with NOM P-value < 0.05 and FDR q-value < 0.25 were considered as significantly enriched, *NOM* nominal, *FDR* false discovery rate.

### Co-expression analysis

Since *XRCC2* was not involved in the malignant progression of glioma through a single cellular mechanism, we speculated that other genes co-express with *XRCC2* in glioma. To test our hypothesis, we used a Pearson analysis to explore the genes co-expressed with *XRCC2* in gliomas and used a circle map to show the top ten and the last ten genes co-expressed with *XRCC2* Additional file 4: Figure S1, Table 3. Combined with the above results, co-expression analysis further demonstrated that *XRCC2* is not involved in the malignant progression of glioma on its own, although it can independently affect the prognosis of glioma.

**Table 3:** Genes co-expressed with *XRCC2*

No.	Gene	Correlation index	*P*-value
1	*LDHD*	− 0.498	7.72E-65
2	*FBXW4*	− 0.49	1.13E-62
3	*MATK*	− 0.483	1.06E-60
4	*CTD*-*2210P24.4*	− 0.475	1.85E-58
5	*SNCG*	− 0.459	4.07E-54
6	*RP11-227B21.2*	− 0.457	1.19E-53
7	*CYP46A1*	− 0.452	1.75E-52
8	*ASPDH*	− 0.446	4.97E-51
9	*SNAI3-AS1*	− 0.442	4.84E-50
10	*NEBL-AS1*	− 0.439	4.32E-49
11	*CENPI*	0.853	1.14E-288
12	*KIF14*	0.852	4.75E-288
13	*MCM8*	0.848	7.06E-282
14	*CKAP2L*	0.844	5.98E-277
15	*BUB1*	0.842	1.97E-274
16	*FAM111B*	0.84	1.21E-272
17	*SGOL2*	0.837	1.89E-268
18	*NCAPG2*	0.831	2.95E-261
19	*ATAD5*	0.83	4.20E-260
20	*RAD51AP1*	0.822	3.46E-251

### Identification of anti-glioma micromolecules targeting XRCC2

**Fig. 6:**
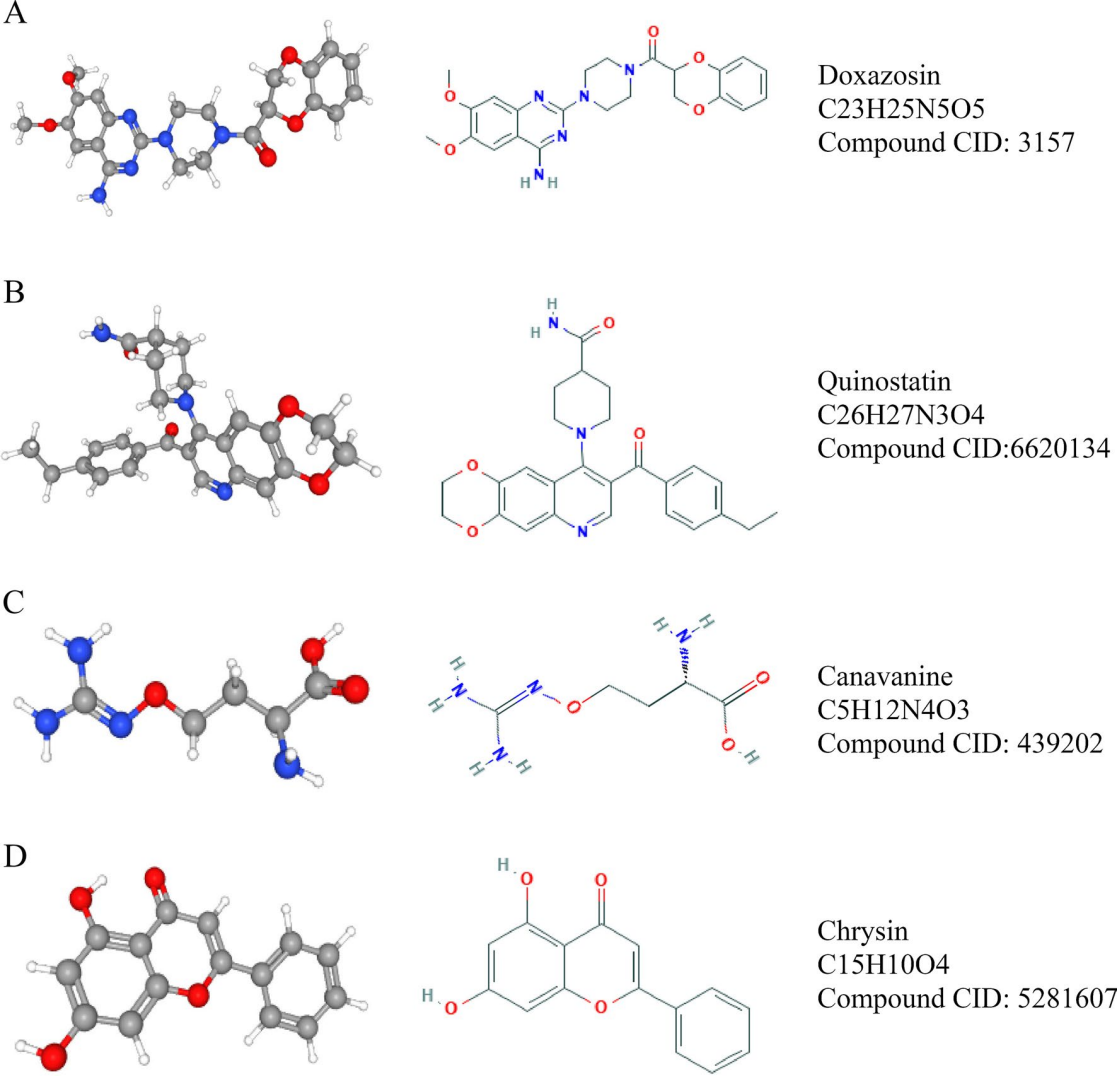
Information regarding small molecules that target *XRCC2*. **A** Doxazosin; **B** Quinostatin; **C** Canavanine; and **D** Chrysin

Based on the role of *XRCC2* in glioma progression, it was necessary to screen for anti-glioma drugs that target *XRCC2*. Therefore, we used the CMap to explore anti-glioma micromolecular drugs that can target *XRCC2*. The results of CMap analysis suggested that doxazosin, quinostatin, canavanine, and chrysin have a high potential for clinical application against glioma by targeting *XRCC2* Table 4. The two-and three-dimensional structures of these four small molecules are shown in Fig. 6.

**Table 4:** Small molecules screened from CMap

No.	CMap name	Enrichment index	*P-*value
1	Doxazosin	− 0.847	0.00101
2	Quinostatin	− 0.963	0.00308
3	Canavanine	− 0.859	0.00563
4	Chrysin	− 0.809	0.0138

*CMap* connectivity map

## Discussion

*XRCC2* is a DNA repair gene that has been found to play an essential role in the development of many cancers (Curtin 2009; Hilbers 2016). Previous reports have suggested that *XRCC2* is a target of microsatellite instability in medulloblastoma (Viana-Pereira 2009); however, no studies have revealed the expression pattern of *XRCC2* in glioma, analyzed the correlation between *XRCC2* and clinical features of glioma, or elucidated the impact of *XRCC2* on the prognosis of glioma patients. This study comprehensively analyzed the expression pattern of *XRCC2* in glioma and found that overexpression of *XRCC2* can reduce the survival rate of glioma patients. Based on the mutual validation of multiple public databases, we identified the possible *XRCC2* signaling pathways involved in the malignant progression of glioma and screened four small-molecule drugs with potential clinical application value.

GEPIA2 integrates the tumor data from TCGA database and the data of normal tissue samples from the GTEx database, and currently, it is the best online analysis tool to explore the levels of gene expression in cancer and normal tissue. In this study, based on the GEPIA2 analysis, we found that the expression level of *XRCC2* in various cancers, including GBM, was significantly higher than that in the corresponding normal tissues. Moreover, this result was validated in a variety of glioma cell lines and tissues based on transcriptome and protein levels Fig. 1. Studies have reported that *XRCC2* affects the efficacy of cancers and participates in the malignant progression of different tumors. For example, silencing *XRCC2* can block the cell cycle and increase the sensitivity of rectal cancer cells to radiotherapy (Qin 2015), while the overexpression *of XRCC2* in colon cancer promotes the proliferation of cancer cells (Xu 2014). In addition, studies have shown that *KIF14*, a co-expressed gene positively associated with *XRCC2*, plays an oncogenic role in numerous cancers (Corson et al. 2005), *FBXW4*, a gene negatively associated with *XRCC2*, acts as a protective factor in metastatic colorectal cancer (Zhang 2020). Therefore, we speculate that *XRCC2* is involved in the malignant progression of glioma as an oncogene and, hence, its overexpression affects the prognosis of patients.

To determine the effect of *XRCC2* overexpression on the prognosis of glioma patients, we performed survival analysis on different data sets, and, as predicted, we found that overexpression of *XRCC2* reduced patient survival. As the occurrence of cancer is dependent on many factors, we used univariate and multivariate analyses to test whether this effect of *XRCC2* on the prognosis of glioma is an independent factor. As we predicted, *XRCC2* was an independent risk factor for poor prognosis in glioma, demonstrated by meta-analysis of multiple datasets. The histological characteristics of glioma play an important role in tumor classification, diagnosis, and treatment (Lapointe et al. 2018; Weller 2015). The results of multivariate analysis in this study suggest that histology is not an independent factor affecting the prognosis of gliomas (P > 0.05). This may be because the interaction between *XRCC2* and histology reduces the effect of histology on the prognosis in multivariate analysis, which further reveals that *XRCC2* can affect other factors to reduce the prognosis of glioma. In addition, the WHO in 2016 included *IDH* mutations and 1p/19q combined deletions into the classification and diagnosis of gliomas (Louis 2016), which indicates that molecular characteristics play an increasingly important role in the diagnosis, treatment, and prognosis evaluation of gliomas. Therefore, we have reason to believe that *XRCC2*, which is highly expressed in gliomas, is an independent risk factor affecting the prognosis of gliomas. Subsequently, it lays the foundation for studying the relationship between *XRCC2* and glioma histology. Moreover, multiple data sets mutually verified and revealed that *XRCC2* has a high diagnostic value in gliomas Figs. 2, 3, and it also increases the credibility of our research results. In addition, previous studies support our findings, reports suggest that overexpression of *XRCC2* in rectal cancer predicts higher malignancy and more lymphatic metastasis and that abnormal overexpression of *XRCC2* predicts poor prognosis in breast cancer patients (Lin 2011; Qin 2015).

It is well known that gliomas with high WHO grade have a poor prognosis and that patients with *IDH* mutation and *1p/19q* co-deletion have a higher survival rate. Therefore, we speculated that the expression level of *XRCC2* in glioma may also be correlated with multiple clinical and molecular characteristics associated with its prognosis. As expected, the results showed that *XRCC2* has a higher expression level in gliomas with higher malignancy and that the expression level of *XRCC2* is lower in patients with *IDH* mutation and *1p/19q* co-deletion than in the corresponding control group. These results indirectly reveal that *XRCC2* participates in the malignant progression of glioma and affects the prognosis of glioma patients.

Moreover, to understand how *XRCC2* participates in the malignant progression of glioma and it actually reduces the survival rate of patients, we performed GSEA analysis on three different datasets and found that the signaling pathways of cell cycle, DNA replication, homologous recombination, and mismatch repair were significantly enriched in all three data sets. These signaling pathways play an essential role in the occurrence and development of cancer. For instance, the cell cycle signaling pathway plays a vital role in the malignant progression of multiple tumors. Studies have found that a variety of anti-glioma drugs act by targeting the cell cycle pathway (Song 2018; Lu 2018; Huang 2016). The dysregulation of DNA replication, one of the fundamental biological processes of the cell, can lead to genomic instability, which in turn promotes the occurrence of cancer (Kitao 2018; Macheret and Halazonetis 2015). The dysfunction of homologous recombination, a mechanism involved in the repair of DSBs, which mainly occurs in the late S-G2 phase of the cell cycle, can result in the process of tumorigenesis (Hoppe et al. 2018); mismatch repair plays a crucial role in maintaining gene stability and provides a new perspective for glioma immunotherapy (Hutchinson 2016; Baretti and Le 2018). These signaling pathways targeted by *XRCC2* indicate that *XRCC2*, as an oncogene, is involved in the occurrence and malignant progression of glioma through multiple pathways, ultimately affecting the prognosis of patients. These results provide a valuable reference for further exploration of molecular therapy for glioma.

To identify small-molecule drugs that can be used to target *XRCC2* against glioma, we explored four small-molecule compounds with potential clinical applications using CMap online tools based on the CGGA RNA-seq dataset: doxazosin, quinostatin, canavanine, and chrysin. Previous studies have shown that doxazosin can inhibit the growth of GBM cells through the *PI3K/AKT* pathway (Gaelzer 2016). Chrysin can inhibit the invasion and migration characteristics of glioma cells through *ERK/NRF2* and inhibit the growth of glioma cells (Wang 2018). Quinostatin and canavanine also exhibit antitumor potential in a variety of tumors (Zhang et al. 2019; Nurcahyanti and Wink 2015). In recent years, the new application of traditional medicine has gradually gained attention and achieved satisfactory results in basic research and clinical applications. For example, atorvastatin, a previously considered anti-lipid drug, can be used to treat chronic subdural hemorrhage (Jiang 2018). Sildenafil was initially used in the treatment of cardiovascular and cerebrovascular diseases and has achieved satisfactory results in the treatment of male sexual dysfunction (Hatzimouratidis 2010). Therefore, we believe that the four small molecule compounds identified by us may soon play an essential role in anti-glioma therapy targeting *XRCC2*.

However, there are still some shortcomings in this study. First, because not all patients from public databases had complete clinical information, some of the glioma clinical characteristics in this study were not mutually validated between different databases. Nevertheless, each dataset contains hundreds of samples, which can reduce the bias to some extent. Second, due to the lack of clinical information, the sample sizes at different time points differed when performing survival analysis, especially in TCGA RNA-seq data. However, when we examined 5-year and 10-year survival rates, patients with high *XRCC2* expression showed lower survival rates than those with low *XRCC2* expression. Therefore, multicenter studies with complete clinical information still need to be continuously conducted and their data need to be incorporated into public databases, providing a complete research platform for glioma researchers worldwide.

## Conclusion

This study pioneered in exploring the expression pattern of *XRCC2* in glioma, clarifying that *XRCC2* can act as an oncogene and reduce the survival rate of glioma patients. We found that *XRCC2* is an independent risk factor for poor prognosis in glioma patients and has a high diagnostic value for glioma prognosis. Furthermore, we found that *XRCC2* can participate in tumorigenesis by affecting cell cycle, DNA replication, homologous recombination, and mismatch repair signaling pathways. In order to reduce the harm caused by *XRCC2* overexpression, based on the CGGA RNA-seq dataset we identified small molecules that target *XRCC2* that have the potential clinical application of improving the status of glioma treatment and the prognosis of glioma patients.

## Supplementary Information

**Additional file 1: Table S1.** Characteristics of patients with glioma based on CGGA RNA-seq data.

**Additional file 2: Table S2.** Characteristics of patients with glioma based on CGGA microarray data.

**Additional file 3: Table S3.** Characteristics of patients with glioma based on TCGA RNA-seq data.

**Additional file 4: Figure S1.** Circle diagram of co-expression analysis results.

## Data Availability

The datasets used and analyzed during the current study are available from the corresponding author on reasonable request.

## Abbreviations

AUC: Area under the receiver operating characteristic
CGGA: Chinese Glioma Genome Atlas
CI: Confidence interval
CMap: Connectivity map
DSBs: DNA double-strand breaks
GBM: Glioblastoma multiforme
GEO: Gene expression omnibus
GEPIA2: Gene expression profiling interactive analysis 2
GSEA: Gene set enrichment analysis
GTEx: Genotype-tissue expression
HA: Human astrocyte
HR: Hazard ration
IDH1: Isocitrate dehydrogenase 1
KEGG: Kyoto encyclopedia of genes and genomes
PRISMA: Preferred Reporting Items for Systematic reviews and Meta-Analyses
ROC: Receiver operating characteristic
RT-qPCR: Real-time quantitative polymerase chain reaction
TCGA: The Cancer Genome Atlas
WHO: World health organization
