# Supplementary material for: Overexpressed XRCC2 as an independent risk factor for poor prognosis in glioma patients
Source: Mol Med. 2021 May 29;27:52. doi: 10.1186/s10020-021-00316-0 (PMC8164800; doi:10.1186/s10020-021-00316-0)
Supplement: Supplementary file 3 — Additional file 3: Table S3. Characteristics of patients with glioma based on TCGA RNA-seq data. [file 10020_2021_316_MOESM3_ESM.docx]

Table S3. Characteristics of patients with glioma based on TCGA RNA-seq data.

| Characteristics |  | Number of cases | Percentages(%) |
| --- | --- | --- | --- |
| Gender | Male | 377 | 57.73 |
|  | Female | 276 | 42.27 |
| Age | <=51 | 394 | 60.34 |
|  | >51 | 259 | 39.66 |
| Grade | WHO II | 238 | 36.45 |
|  | WHO III | 256 | 39.20 |
|  | WHO IV | 159 | 24.35 |
