# Supplementary figures and images for: Overexpressed XRCC2 as an independent risk factor for poor prognosis in glioma patients
Source: Mol Med. 2021 May 29;27:52. doi: 10.1186/s10020-021-00316-0 (PMC8164800; doi:10.1186/s10020-021-00316-0)

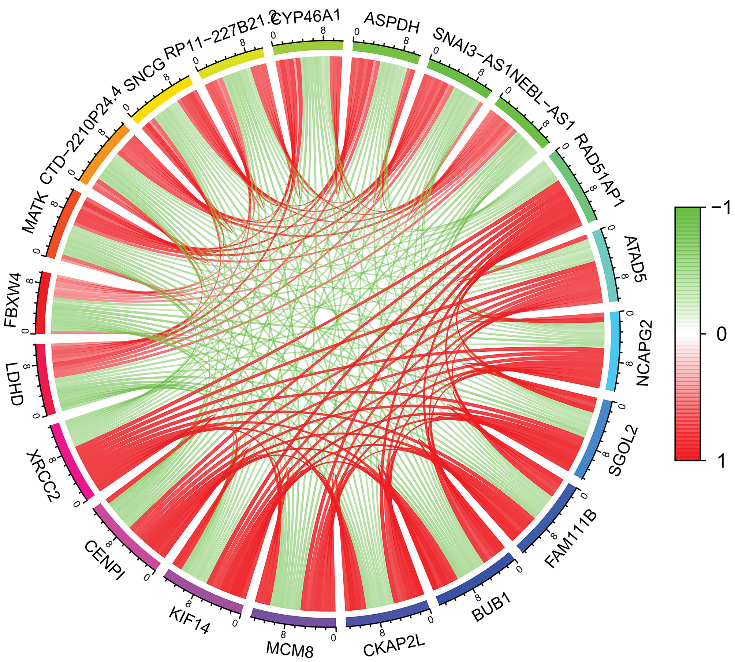

Supplement: Supplementary file 4 — Additional file 4: Figure S1. Circle diagram of co-expression analysis results. [file 10020_2021_316_MOESM4_ESM.docx]
